# Ivacaftor-Mediated Potentiation of ABCB4 Missense Mutations Affecting Critical Motifs of the NBDs: Repositioning Perspectives for Hepatobiliary Diseases

**DOI:** 10.3390/ijms24021236

**Published:** 2023-01-08

**Authors:** Jean-Louis Delaunay, Ahmad Elbahnsi, Alix Bruneau, Claire Madry, Anne-Marie Durand-Schneider, Anne Stary, Chantal Housset, Jérémie Gautheron, Isabelle Callebaut, Tounsia Aït-Slimane

**Affiliations:** 1 Sorbonne Université, Inserm, Centre de Recherche Saint-Antoine (CRSA), Institute of Cardiometabolism and Nutrition (ICAN), 75012 Paris, France; 2 Sorbonne Université, Muséum National d’Histoire Naturelle, UMR CNRS 7590, Institut de Minéralogie, de Physique des Matériaux et de Cosmochimie, IMPMC, 75005 Paris, France; 3 Department of Hepatology & Gastroenterology, Charité Universitätsmedizin Berlin, 13353 Berlin, Germany; 4 Assistance Publique-Hôpitaux de Paris, Hôpital Saint-Antoine, Centre de Référence des Maladies Rares Maladies Inflammatoires des Voies Biliaires et Hépatites Auto-Immunes & Service d’Hépatologie, 75012 Paris, France

**Keywords:** bile secretion, genetic liver disease, PFIC3, ABC transporter, potentiators, ivacaftor

## Abstract

ABCB4 (ATP-binding cassette subfamily B member 4) is a hepatocanalicular floppase involved in biliary phosphatidylcholine (PC) secretion. Variations in the ABCB4 gene give rise to several biliary diseases, including progressive familial intrahepatic cholestasis type 3 (PFIC3), an autosomal recessive disease that can be lethal in the absence of liver transplantation. In this study, we investigated the effect and potential rescue of ten ABCB4 missense variations in NBD1:NBD2 homologous positions (Y403H/Y1043H, K435M/K1075M, E558K/E1200A, D564G/D1206G and H589Y/H1231Y) all localized at the conserved and functionally critical motifs of ABC transporters, six of which are mutated in patients. By combining structure analysis and in vitro studies, we found that all ten mutants were normally processed and localized at the canalicular membrane of HepG2 cells, but showed dramatically impaired PC transport activity that was significantly rescued by treatment with the clinically approved CFTR potentiator ivacaftor. Our results provide evidence that functional ABCB4 mutations are rescued by ivacaftor, paving the way for the repositioning of this potentiator for the treatment of selected patients with PFIC3 caused by mutations in the ATP-binding sites of ABCB4.

## 1. Introduction

The adenosine triphosphate (ATP)-binding cassette, sub-family B, member 4 (ABCB4), also known as multidrug resistance 3 (MDR3), is a hepatocanalicular floppase involved in biliary phosphatidylcholine (PC) excretion [1]. PC forms mixed micelles with co-secreted bile salts and cholesterol by ABCB11 and ABCG5/G8, respectively. The formation of bile-acid/PC mixed micelles is critical to reduce the detergent activity of bile acids and to prevent the formation of cholesterol gallstones. In the absence of PC due to ABCB4 defects, the hepatocytes and cholangiocytes that line the biliary tree are exposed to the damaging detergent action of free bile acids, leading to inflammation and cholestasis (Reviewed by Reichert and Lammert [2]). The most severe liver disease related to dysfunctional ABCB4 is progressive familial intrahepatic cholestasis type 3 (PFIC3), which is characterized by the early onset of persistent cholestasis that progresses to cirrhosis and liver failure before adulthood and most often requires liver transplantation [3]. Less severe diseases are low-phospholipid-associated cholelithiasis (LPAC) syndrome, which occurs in young adults [4], and intrahepatic cholestasis of pregnancy [5]. Up to now, about 400 distinct disease-causing ABCB4 variants have been reported. A challenge is to find pharmacological treatments for the severe forms of these diseases. We have unraveled several mechanisms by which ABCB4 missense variations cause diseases, and proposed a functional classification of these variations based on their impact on the traffic, activity or stability of the protein [6]. 

The resolution of the ABCB4 three-dimensional (3D) structure [7] confirmed that ABCB4 has the characteristics of type IV ABC transporters [8]. It is composed of two membrane-spanning domains (MSDs), each consisting of six transmembrane helices, which allow PC translocation, and two nucleotide-binding domains (NBDs) that contain several conserved motifs involved in ATP binding and hydrolysis. Structural and site-directed mutagenesis studies carried out on various ABC transporters have elucidated the role of several residues in each of these functionally critical motifs, notably (i) the Walker A motif, which binds the alpha- and beta-phosphates of ATP; (ii) the Walker B motif, which provides a conserved catalytic glutamate; (iii) the A-loop, which includes a residue with an aromatic side chain interacting with the adenine ring of ATP; (iv) the D-loop, which is involved in ATP hydrolysis; (v) the H-loop or a switch histidine, which contributes to the catalytic reaction by stabilizing the transition-state geometry; (vi) the Q-loop, which connects the catalytic site and the helical subdomain of the NBD and interacts with bound ATP, and (vii) the LSGGQ signature, which is a specificity of ABC transporters and is involved in the pinning and orientation of ATP during hydrolysis (More details about the mechanistic of these conserved motifs can be found in the review by Kroll et al. [9]). 

We previously showed that five disease-causing mutations, four of which are localized in the signature motifs of NBD1 and NBD2 and one at the Walker A of NBD2 of ABCB4, resulted in a defect in ABCB4 function, which could be rescued by the clinically approved CFTR potentiator, ivacaftor [10]. The beneficial effect of ivacaftor was subsequently reported to rescue functional mutants in the ATP-binding sites of another type IV ABC transporter, the bile acid transporter ABCB11 [11]. 

To gain insight into the molecular mechanism of ABCB4 potentiation, we identified and functionally characterized ten variants in NBD1:NBD2 homologous positions. All of them are localized within the conserved and functionally critical ABCB4 motifs, six of which are mutated in PFIC3 or LPAC syndrome patients. We also evaluated the effect of ivacaftor on the PC transport activity of the ten mutants. By combining structure analysis and in vitro studies, we found that all ten mutants were normally processed and localized at the plasma membrane but showed dramatically impaired PC transport activity that was significantly rescued by treatment with the CFTR potentiator ivacaftor. 

## 2. Results

### 2.1. ABCB4 Variations in Critical Motifs of the NBDs and Liver Diseases

**Figure 1:**
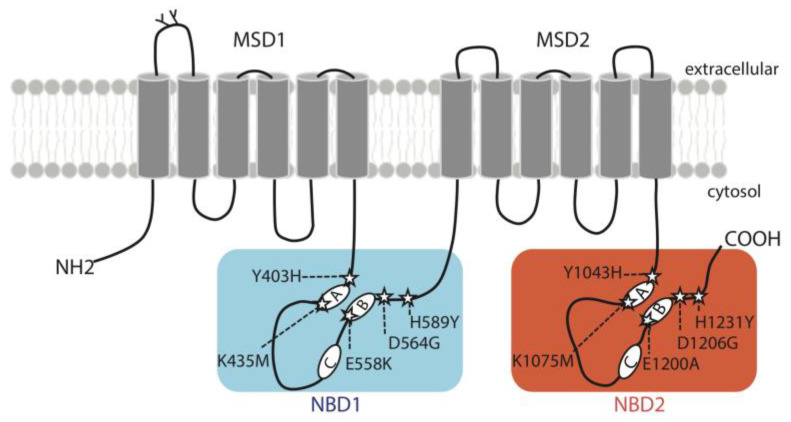
Topology diagram of ABCB4 illustrating the location of the variants studied here. The five mutations of the conserved motifs in NBD1 (Y403H, K435M, E558K, D564G and H589Y) are boxed on a blue background, and mutations of corresponding residues in NBD2 (Y1043H, K1075M, E1200A, D1206G and H1231Y) are boxed on an orange background. A: Walker A motif, B: Walker B motif, C: signature motif for ABC transporters, MSD: membrane-spanning domain, NBD: nucleotide-binding domain.

The distribution of ABCB4 variations studied here are shown in Figure 1. They affect homologous positions that all belong to highly conserved motifs of ABCB4 NBD1 and NBD2 (Supplementary Figure S1), including the A-loops (Y403H in NBD1; Y1043 in NBD2), the Walker A motifs (K435M in NBD1; K1075M in NBD2), the Walker B motifs (E558K in NBD1; E1200A in NBD2), the D-loops (D564G in NBD1; D1206G in NBD2) and the H-loops (H589Y in NBD1; H1231Y in NBD2). Of the ten variants studied, six (Y403H, E558K, D564G, H589Y, E1200A and H1231Y) were identified in patients and four (K435M, Y1043H, K1075M and D1206G) are theoretical variants. Y403H, H1231Y with homozygous status and E558K, D564G with compound heterozygous status were detected in PFIC3 patients [3,12,13]. H589Y and E1200A with heterozygous status were detected in patients with LPAC syndrome [14,15]. The main characteristics of the patients are shown in Table 1.

### 2.2. The Structure of ABCB4 NBDs and In Silico Predictions of the Impact of the Variants

**Figure 2:**
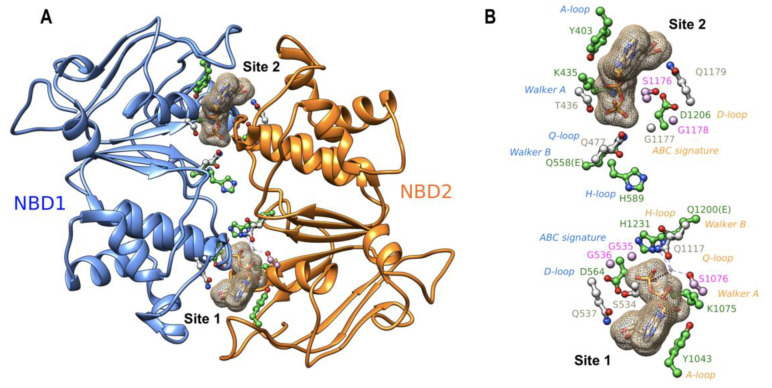
(**A**) Ribbon representation of the experimental 3D structure of the human ABCB4 NBD1:NBD2 dimer (PDB: 6S7P), viewed from the membrane towards the cytosol. The amino acids of the conserved motifs at the interface between the two domains are highlighted in a ball-and-stick representation. The two ATP molecules are represented as surface meshes. (**B**) The same view as in panel A, highlighting only the amino acids of the conserved motifs. Amino acids whose mutations were analyzed in this study are colored in green, those studied previously are in pink, and the other ones are in gray. The magnesium ion bound to site 1 ATP is also shown. Motif names are colored according to their belonging to NBD1 (blue) or NBD2 (orange).

A ribbon view of the 3D structure of the NBD1:NBD2 head-to-tail dimer, viewed from the membrane, is displayed in Figure 2A, while the main amino acids involved in the two composite ATP binding sites are depicted in Figure 2B. ATP binding site 1 is formed by residues belonging, on the one hand, to the NBD2 A-loop, Walker A and Walker B motifs and H-loop and, on the other hand, to the NBD1 ABC signature and D-loop. ATP binding site 2 is formed by residues belonging to the NBD1 A-loop, Walker A and Walker B motifs and H-loop and to the NBD2 ABC signature and D-loop. Although ABCB4 contains two consensus ATP-binding sites, a structural asymmetry was observed in the cryo-EM 3D structure, with a magnesium ion only observed at site 1 [7]. This ion mediates strong contacts between the beta and gamma phosphates of ATP and NBD2 Walker A and Q-loop residues and helps to orientate ATP in site 1 (Figure 2A,B). Asymmetry is also observed for the position of the histidine of H-loops, with NBD2 H1231 also well orientated towards site 1 to form a dyad with the Walker B catalytic base [7], whereas NBD1 H589 shifts towards the NBD1:NBD2 interface (Figure 2B). As in ABCB1 [17], ATP molecules in the two sites are capped by the A-loops, with the conserved tyrosines (Y403 and Y1043) stacking adenine and making H-bonds with Walker A amino acids on one side of the site. The aspartic acids of the D-loops are positioned similarly. These amino acids were proposed to coordinate the attacking water in the hydrolysis reaction and participate in the modulation of the hydrolysis competent state [18]. As all the amino acids studied here form critical bonds or contacts through their side chains with ATP, magnesium ions or water molecules, the studied mutations are predicted to impair ATP binding and hydrolysis.

### 2.3. Localization of ABCB4 Mutants in HepG2 and HEK293 Cells

**Figure 3:**
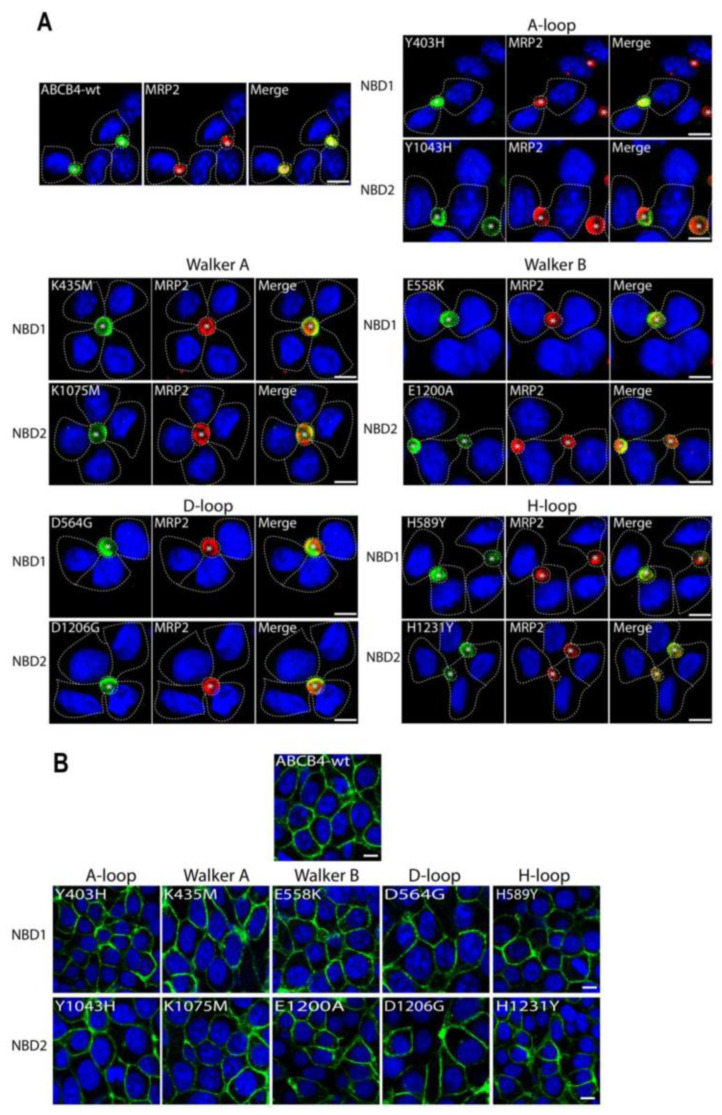
Localization of ABCB4-wt and mutants in HepG2 and HEK293 by immunofluorescence and confocal microscopy. (**A**) HepG2 cells transiently expressing ABCB4-wt or mutants of the conserved motifs (A-loop, Walker A, Walker B, D-loop and H-loop) in NBD1 and NBD2 were fixed and permeabilized, and processed for immunofluorescence using the anti-ABCB4 (P3-II-26) and anti-MRP2 (M2-I-4) monoclonal antibodies, followed by goat anti-IgG2b Alexa Fluor 488- and goat anti-IgG1 594-conjugated secondary antibodies, and visualized by confocal microscopy. In transfected cells, ABCB4-wt and all mutants are exclusively detected at the canalicular membrane and colocalized with endogenously expressed MRP2; yellow denotes the colocalization of both proteins in merged images. Nuclei are stained in blue with Draq5. Transfected cells are indicated by dashed lines. Bile canaliculi are indicated by asterisks. Bars = 10 µm. (**B**) The localization of ABCB4-wt or the mutants in stably transfected HEK293 cells was assessed by indirect immunofluorescence using anti-ABCB4 antibodies as in (**A**). The data show that as in HepG2 cells, ABCB4-wt and all mutants were expressed at the plasma membrane. Bars = 10 µm.

To functionally characterize the variants identified in the critical motifs of the ABCB4 NBDs, we first studied their impact on ABCB4 subcellular distribution. The mutated complementary DNAs reproducing the ABCB4 variations were transfected in polarized HepG2 cells and nonpolarized epithelial HEK293. HepG2 cells derive from a human hepatocellular carcinoma and form neo-bile canaliculi in culture and allow localization studies, whereas HEK293 cells are suitable for studies of transport activity. It is worthy of note that, in our experimental conditions, no endogenous ABCB4 was detected in either cell line. The localization of the mutants was compared to that of ABCB4-wt after transient and stable transfection in HepG2 and HEK293 cells, respectively. Forty-eight hours after transfection, HepG2 cells were fixed, permeabilized and stained for ABCB4, in addition to MRP2 as a canalicular marker [10]. Confocal microscopy showed that ABCB4-wt was exclusively detected at the canalicular membrane where it co-localizes with MRP2 (Figure 3A). Similarly to ABCB4-wt, all the mutants displayed canalicular localization and colocalized with MRP2 (Figure 3A). In HEK293 cells, the subcellular localization of the mutants was studied after the selection of stable cell populations. As in HepG2 cells, all of the mutants were localized exclusively at the plasma membrane (Figure 3B). These observations indicate that all the mutants of the conserved motifs of the ABCB4 NBDs did not impair the intracellular trafficking and the plasma membrane localization of ABCB4.

### 2.4. Expression and Processing of ABCB4 Mutants

**Figure 4:**
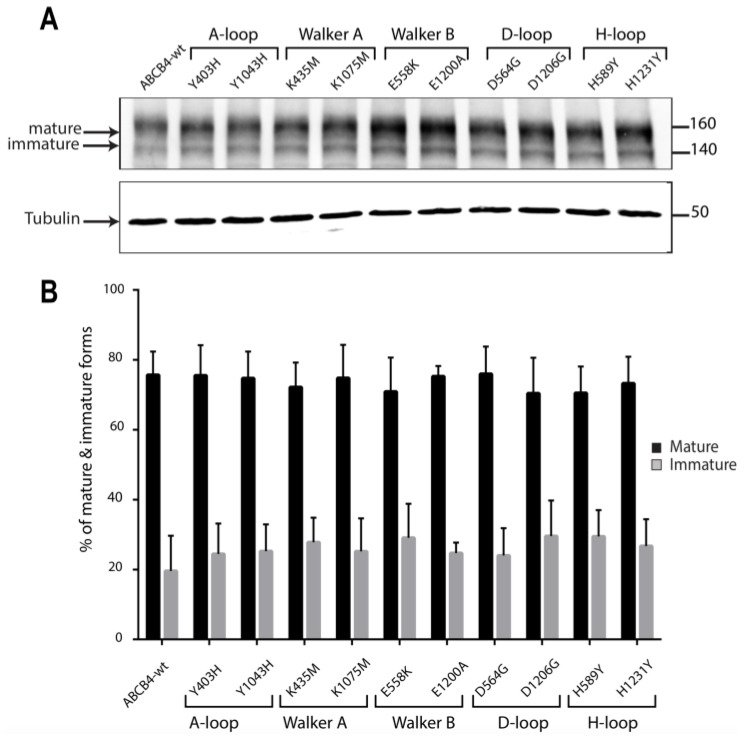
(**A**) Representative Western blot of the expression levels of the mutants with respect to ABCB4-wt. The expression and the processing of ABCB4-wt and the mutants was examined by Western blot analysis of whole-cell lysates from stably transfected HEK293 cells. ABCB4 expression was detected following SDS-PAGE and immunoblotting with the P3-II-26 antibody. Tubulin was used as a loading control. Molecular masses are indicated on the right (in kDa). Presented data were cropped from full immunoblots shown in supplementary Figure S2. (**B**) Biological replicates were quantified by densitometry. The mature and immature bands were separately quantified on gels, and their relative amounts were calculated. Results are the means (±SD) of at least three independent experiments.

The expression and the processing of the mutants were assessed by Western blot analyses and compared to that of ABCB4-wt. As shown in the representative blot (Figure 4A), ABCB4-wt was expressed as a major mature band migrating with an apparent molecular weight of 160 kDa and a minor immature band at 140 kDa, as previously reported [19]. The ten mutants displayed the same pattern of migration; they were found essentially under the slow-migrating 160 kDa form (Figure 4A). The quantification of replicate data sets confirmed that the expression profile of all mutants was indistinguishable from that of ABCB4-wt (Figure 4B). These results indicate that the ten mutants of the conserved motifs of the ABCB4 NBDs did not impair the expression and the maturation of ABCB4. 

### 2.5. PC Secretion Activity of ABCB4 Mutants and the Effect of Ivacaftor

**Figure 5:**
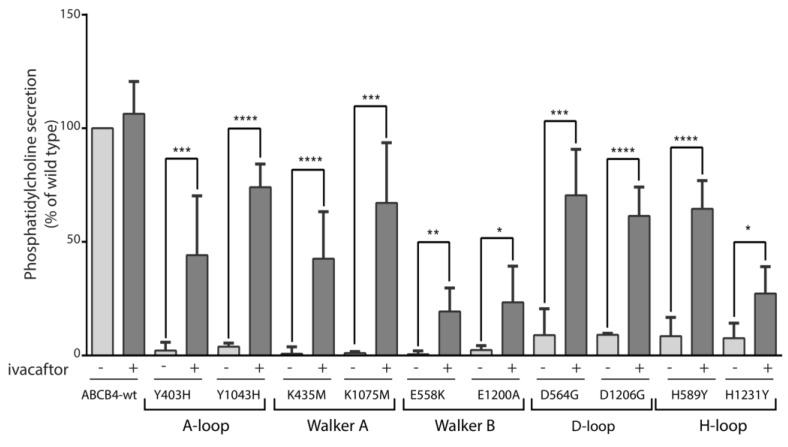
PC secretion activity of ABCB4-wt and the mutants and response to ivacaftor. HEK293 cells were transiently transfected with plasmids encoding ABCB4-wt or the indicated mutants, and PC secretion was measured after 24 h in the absence (−) or presence (+) of 10 µmol/L of ivacaftor. PC secretion was normalized to the expression level of the mature form of the respective protein (ABCB4-wt or mutants) and expressed as a percentage of the secretion activity of ABCB4-wt. Results are the means (±SD) of at least six independent experiments performed in triplicate. * *p* < 0.05; ** *p* < 0.01; *** *p* < 0.001; **** *p* < 0.000.

As all the mutants were located in functionally critical motifs of ABCB4, we hypothesized that their PC secretion activity would be impaired. Furthermore, as all mutants were expressed exclusively at the plasma membrane, it seems obvious that their function was altered, which strongly supports our hypothesis. The PC secretion activity of the mutants was examined in transiently transfected HEK293 cells and compared to that of ABCB4-wt. We observed that, as expected, no PC secretion activity could be measured for either of these mutants (Figure 5). These results indicated that mutations in the critical motifs of ABCB4 impaired its PC secretion activity. Previously, we have shown that the PC secretion defect of five disease-causing mutations, four of which are located in the ABC signature motifs of NBD1 and NBD2 and one in the Walker A of NBD2 of ABCB4 could be rescued by the clinically approved CFTR potentiator ivacaftor [10]. We then wondered whether the potentiating effect of ivacaftor could be extended to all mutants located in the functionally critical motifs of ABCB4. For this purpose, we tested the effect of ivacaftor on the ten mutants. HEK293 cells transiently expressing ABCB4-wt or the mutants were treated with 10 µmol/L of ivacaftor for 24 h, as previously described [10]. As shown in Figure 5, treatment with ivacaftor rescued the mutants Y403H/Y1043H (up to 44% and 70% of WT, respectively), K435M/K1075M (up to 42% and 67% of WT, respectively), E558K/E1200A (up to 20% and 23% of WT, respectively), D564G/D1206G (up to 70% and 61% of WT, respectively) and H589Y/H1231Y (up to 64% and 27% of WT, respectively). These effects on PC secretion activity are in line with those previously reported for ABCB4 missense mutations that reside in the LSGGQ signature motifs of NBD1 and NBD2 and the Walker A of the NBD2 [10], and indicate that the potentiating effect of ivacaftor can be extended to all mutants located in ATP-binding sites of ABCB4.

## 3. Discussion

In the present study, we examined the effects of ten ABCB4 missense variations (Y403H/Y1043H, K435M/K1075M, E558K/E1200A, D564G/D1206G and H589Y/H1231Y) that reside in the NBD1:NBD2 homologous positions within the highly conserved motifs of ABC transporters, which are involved in ATP binding and hydrolysis. Among the ten mutations studied, six were identified in patients. We show that, although correctly targeted to the canalicular membrane, all of the mutants significantly impaired the ability of ABCB4 to secrete PC from cells. According to our functional classification, all the variants belong to class III variations [6]. Interestingly, the functional defect displayed by the ten mutants was successfully rescued by the clinically approved CFTR potentiator, ivacaftor.

All of the mutants affect highly conserved motifs within the nucleotide binding sites of ABCB4 and other ABC transporters. Y403/Y1043 were located at equivalent positions of NBD1 and NBD2 of ABCB4, respectively, and they form the A-loop that interacts with the adenine ring of ATP. Several studies have been conducted to assess the effects of mutating this aromatic residue in several ABC transporters (for review see Ambudkar et al., 2006 [20]). The Y16S mutation in the HisP subunit of the bacterial histidine permease was shown to prevent the binding of ATP and its transport function [21]. Kim et al., 2006, showed that the replacement of these aromatic residues on MDR1 (Y401, Y1044) with non-aromatic residues results in the loss of ATP binding and hydrolysis and also affects its transport function [22]. The Y403H variation was identified in a homozygous patient who was diagnosed as PFIC3 at the age of 3 months (no. 1, Table 1) [12]. The immunohistochemical staining of ABCB4 in liver biopsies from this patient previously showed the presence of ABCB4 at the canalicular membrane in at least 60% of hepatocytes [23]. In HUH28 cells transfected with the Y403H mutant, Degiorgio et al. showed a defect in phosphatidylcholine secretion, although the cell surface expression of the mutant was comparable to that of wild-type ABCB4 [24]. In agreement with this observation, we found that Y403H and Y1043H mutants were detected at the canalicular membrane of transfected HepG2 cells, but displayed major activity defects. K435/K1075 are part of the Walker A motifs of NBD1 and NBD2 of ABCB4, respectively. The amino acids of the Walker A motif (GCGKS) are critical for the binding of ATP as they form the phosphate binding loop. Our results are in agreement with those of Morita et al. who showed that the substitution of the lysine by methionine in both NBDs resulted in a defect in PC transport by ABCB4 [16]. In another study, Andress et al. showed that the variant K435T, identified in a patient with Biliary cirrhosis [25], localizes to the plasma membrane of HEK293T cells but lacks PC secretion activity [26]. The residues E558/E1200 are part of the extended Walker B motifs of NBD1 and NBD2 of ABCB4, respectively. The combined mutation of these carboxylate residues in both NBDs strongly reduce the ATPase activity of ABC transporters [27]. The E558K variation was identified in a compound heterozygous patient with PFIC3 clinical phenotype (no. 3, Table 1) [12]. The E1200A variation was identified in a heterozygous patient with LPAC syndrome (no. 8, Table 1) [15]. Our data are in line with those previously reported by two studies, showing that the mutation of glutamate to glutamine in both Walker B motifs (E558Q/E1200Q) resulted in the absence of PC secretion in HEK293 cells [7,28], indicating that the PC secretion activity of ABCB4 was dependent on ATP hydrolysis. The aspartate residues D564/D1206 belong to the D-loop of NBD1 and NBD2 of ABCB4, respectively. The D564G variation was identified in a heterozygous patient with PFIC3 (no. 4, Table 1) [3]. The function of this conserved motif (SALD) has been investigated in several studies. In the *Escherichia coli* ABC transporter MsbA, substitution of the aspartate to glycine (D512G) resulted in the lack of cell viability [29]. In the sulfonylurea receptor SUR1, substitution of the aspartate by a cysteine interferes with the gating of the associated Kir6.2 channel [30]. In the ABC transporter associated with antigen processing (TAP), substitution of the conserved aspartate to alanine leads to a decrease in the dimerization affinity of NBDs and a transformation of the unidirectional active transport into a passive bidirectional transport [31]. In line with these observations, we found that, although correctly targeted to the canalicular membrane of HepG2 cells, D564G and D1206G displayed a major activity defect. The ABCB4 mutants H589Y/H1231Y affect equivalent residues in the H-loop of NBD1 and NBD2, respectively. Equivalent histidines have been shown to be essential for ATP hydrolysis in various ABC transporters. Mutations of the conserved histidine (H211D, H211Y and H211R) in HisP [21] and H192R in Malk [32] and H662A in HlyB [33] resulted in the loss of ATPase activity and the transport function. However, an exception has been reported for the yeast ABC transporter Pdr5. Indeed, Ernst et al. have shown that mutation of the histidine 1068 to alanine (H1068A) had no effect on ATP hydrolysis, but abolished rhodamine transport, while leaving the transport of other substrates unaffected [34]. The H589Y mutation was identified in a heterozygous patient with LPAC syndrome (no. 5, Table 1) [14]. The H1231Y mutation was identified in a homozygous patient who was diagnosed as PFIC3 at the age of 4 years (no. 10, Table 1) [13]. The staining of ABCB4 in a liver biopsy from this patient previously showed normal localization of ABCB4 at the canalicular membrane of hepatocytes, which had suggested that H1231Y mutation did not affect the targeting of the protein at the plasma membrane, but rather could be the cause of a function defect [13]. Consistent with this observation, we found that both H589Y and H1231Y mutants were detected at the canalicular membrane of HepG2 cells but in a completely inactive form. As the ten residues studied are located in conserved motifs involved in NBDs dimerization and ATP binding and/or hydrolysis, it is not surprising that their substitution results in a defect in ABCB4 PC transport function. Because we have previously shown the efficacy of the clinically approved CFTR potentiator ivacaftor on five function-defective mutants located in the ATP-binding sites of ABCB4 [10], it was tempting to suggest that the potentiating effect of this molecule could be extended to all mutants that affect these sites. Indeed, we show here that the functional defect of the ten mutants was successfully rescued by ivacaftor, although the efficiency of the correction varies from one mutant to another. The weakest effect is observed for mutations of the Walker B catalytic glutamate (E558K/E1200A), consistent with the critical role of this residue in ATP hydrolysis (see above). Interestingly, a difference in the effect is also observed relative to the mutations of the homologous histidine of the H-loop (H589Y/H1231Y), which might be related to the asymmetry which was observed for the position of these residues relative to the ATP-binding sites in the cryo-EM 3D structure of ABCB4 [7]. Ivacaftor is a highly effective drug clinically approved to treat cystic fibrosis patients carrying mutations affecting CFTR channel gating that works by increasing channel open probability [35,36]. Ivacaftor enhances the ATP-independent activity of wild-type CFTR to a similar extent as its effect on G551 D mutation, abolishing responsiveness to ATP, but also increases the open time of wild-type CFTR in an ATP-dependent-manner [37,38]. The fact that ivacaftor has been shown to be effective on other members of the ABC transporter family [10,11,39] suggests a common mechanism of action. The mechanism of rescue by ivacaftor is not yet clear, but is likely to be complex. The binding site of ivacaftor on CFTR was examined by different methods, and several binding sites have been proposed. Using cryo-EM, a binding site considered as specific to CFTR has been identified at the protein-lipid interface within the membrane-spanning domains, involving the transmembrane helices TM8, TM4 and TM5 [40]. This is consistent with the independence of ivacaftor action from ATP hydrolysis or NBD dimerization [37,38]. However, no major change in the overall 3D structure of CFTR occurred when comparing the ivacaftor-bound and unbound CFTR structures, leaving the question of the potentiation mechanism still open. Other ivacaftor-binding sites have been proposed for CFTR at the interface between Membrane-Spanning domain 2 and NBD1 by two different studies, one based on Hydrogen-deuterium exchange coupled with mass spectrometry [41], and the other on the use of two photoactivable probe analogs of ivacaftor on biological membranes [42]. Whether this/these binding site(s) exist(s) on ABCB4 and other ABC transporters remains to be established. A further interesting observation was that ivacaftor was identified as a substrate of P-gp (ABCB1) [43], suggesting that it can bind directly to ABCB1. Indeed, a binding site of ivacaftor, which seems to be absent on CFTR, was identified in the substrate-binding pocket of ABCB1 [44]. As ABCB4 shares 76% sequence identity with ABCB1, it is tempting to speculate that these two transporters may share the same ivacaftor binding site. However, these speculations remain to be tested experimentally. 

In conclusion, the results obtained in this study reinforce our previous findings on the efficacy of ivacaftor on ABCB4 class III mutants, and support the suggestion that CFTR potentiators could be useful, and that therapeutics in patients with ABCB4 function deficiency are caused by mutations in the ATP-binding sites. 

## 4. Materials and Methods

### 4.1. Patients

Six patients, including four with PFIC3 and two with LPAC syndrome were included in the present study (Table 1). An ABCB4 gene analysis was performed as previously described [6].

### 4.2. Antibodies and Reagents

Mouse monoclonal anti-ABCB4 (clone P3-II-26) and anti-MRP2 (multidrug resistance-associated protein 2; clone M2-I-4) antibodies were purchased from Enzo Life Sciences (Villeurbanne, France); and anti-α-tubulin (clone 1E4C11) was sourced from ProteinTech (Manchester, United Kingdom). Alexa Fluor-labeled secondary antibodies, the DRAQ5 fluorescent probe and culture media came from ThermoFisher (Cergy-Pontoise, France), and peroxidase-conjugated secondary antibodies were acquired from Rockland Immunochemicals (Gilbertsville, PA, USA). The ECL-Prime detection kit was from VWR (Courtaboeuf, France). The transfection reagents Turbofect and JetPrime were purchased from Thermo-Fisher Scientific, (Saint-Herblain, France) and Ozyme (Saint-Cyr-l’Ecole, France), respectively. Ivacaftor (VX-770) came from Clinisciences (Nanterre, France). All other reagents were obtained from Sigma-Aldrich (Lyon, France).

### 4.3. The 3D Structure Analysis

Three-dimensional structures were visualized with the UCSF Chimera package [45].

### 4.4. DNA Constructs and Mutagenesis

The construction of the human wild-type (wt) ABCB4 (ABCB4-wt), isoform A (NM_000443.3) in the pcDNA3 vector was reported [19]. pcDNA3-ABCB4-wt was used as a template to introduce the substitutions Y403H, K435M, E558K, D564G, H589Y, Y1043H, K1075M, E1200A, D1206G, and H1231Y by site-directed mutagenesis using the Quik-Change II XL mutagenesis kit (Agilent Technologies, Massy, France). DNA primers used for ABCB4 mutagenesis were acquired from Invitrogen-Life Technologies and are listed in Supporting Table S1. All constructs were verified for the introduction of the substitutions and the absence of additional mutations by automated sequencing of the entire cDNA. 

### 4.5. Cell Culture and Transfections

Human Embryonic Kidney HEK-293 (ATCC^®^-CRL-1573^TM^) cells and Human hepatocellular carcinoma HepG2 (ATCC^®^-HB-8065^TM^) cells were obtained from ATCC (Manassas, VA, USA). They were grown at 37 °C in Dulbecco’s Modified Eagle’s Medium (DMEM), as previously reported [6]. Transfections with plasmids encoding ABCB4-wt or the mutants were performed using Turbofect at a ratio of reagent:DNA of 2:1 for HEK-293 cells, and JetPrime at a ratio of reagent:DNA of 2:1 for HepG2 cells, according to the manufacturer’s instructions and as previously described [6]. Stable expression in HEK-293 cells was obtained by selection with 400 µg/mL of G-418 sulfate (GE Healthcare, Chicago, IL, USA) for three weeks. Cells were subsequently grown in the presence of 100 µg/mL of G-418. For the experiments with HEK-293 cells, plates were precoated with 100 µL poly-L-lysine for 1 h at RT. 

### 4.6. Immunofluorescence Staining and Laser Scanning Confocal Microscopy

Stably transfected HEK-293 cells or transiently transfected HepG2 cells were grown on glass coverslips and fixed with methanol at −20 °C. Incubations with monoclonal primary and secondary antibodies were performed as described [10]. Nuclei were stained with DRAQ5. Images of the stained cells were obtained using a Leica TCS-SP2 laser scanning spectral system attached to a DMR inverted microscope equipped with a 63/1.4 immersion objective. Digital images were analyzed using the online ScanWare software and processed with ImageJ and Photoshop software.

### 4.7. Electrophoresis and Western Blot Analysis

Transfected cells were washed with phosphate-buffered saline (PBS) and lysed at 4 °C for 30 min in TNE buffer (20 mM Tris HCl, 150 mM NaCl, 1 mM EDTA, pH 7.4) containing 1% (*w*/*v*) Triton X-100 in the presence of a protease inhibitor cocktail. Lysates were centrifuged at 12,000× *g* for 10 min to remove insoluble materials. SDS-PAGE on 7.5% (*w*/*v*) polyacrylamide gels and western blotting were performed as previously described [10]. Blots were probed with anti-ABCB4 and anti-α-tubulin used as a loading control. The development of peroxidase activity was performed with the ECL prime western blotting detection reagent. Images were acquired with Ibright imaging systems and signal intensities were quantified using Ibright analysis software.

### 4.8. Measurement of ABCB4-Mediated Phosphatidylcholine Secretion 

HEK293 cells were seeded on poly-lysine-precoated six-well plates at a density of 1.3 × 10^6^ cells/well. Six hours after seeding, the cells were transiently transfected with 1 µg of ABCB4-encoding plasmids using Turbofect, following the manufacturer’s instructions. Twenty-four hours post-transfection, cells were washed twice with Hanks’ balance salt solution, and the medium was then replaced by phenol red-free Dulbecco’s modified Eagle’s medium containing 0.5 mmol/L of sodium taurocholate and 0.02% fatty-acid-free BSA in the presence or absence of 10 µmol/L of ivacaftor as previously described [10]. Media were collected after 24 h. The measurement of PC content in the collected media was performed as described [46]. Results were normalized to the expression levels of ABCB4, which were quantified from immunoblots obtained from the corresponding cell lysates.

### 4.9. Statistics

Data were analyzed using GraphPad Prism 7.00 (La Jolla, CA, USA) and are presented as means ± SD. Statistical analyses were performed using the Student’s *t*-test, with a *p* value < 0.05 considered to be significant.

## Figures and Tables

**Table 1:** Characteristics of Patients with ABCB4 Variations.

Nucleotide Variant	Amino Acid Variant	Zygosity	Localization	Disease	Reference
c. 1207 T>C	Y403H	Ho	AL/NBD1	PFIC3	[12]
c. 1304 A>T	K435M	-	WA/NBD1	TV	[16]
c. 1672 G>A	E558K	CHE/G723E+A1193T	WB/NBD1	PFIC3	[12]
c. 1691 A>G	D564G	HE	DL/NBD1	PFIC3	[3]
c. 1765 C>T	H589Y	HE	HL/NBD1	LPAC	[14]
c. 3127 T>C	Y1043H	-	AL/NBD2	TV	This study
c. 3224 A>T	K1075M	-	WA/NBD2	TV	[16]
c. 3599 A>C	E1200A	HE	WB/NBD2	LPAC	[15]
c. 3617 A>G	D1206G	-	DL/NBD2	TV	This study
c. 3691 C>T	H1231Y	HO	HL/NBD1	PFIC3	[13]

Nucleotide variant corresponds to the complementary DNA of the NM_000443.3 (ABCB4, transcript variant A, messenger RNA). Abbreviations: HO, homozygous; HE, heterozygous; CHE, compound heterozygous; AL, A-Loop; DL, D-Loop; HL, H-Loop; WA, Walker A; WB, Walker B; NBD, nucleotide-binding domain; TV, theoretical variant.

## Data Availability

The datasets generated and analyzed during the current study are available from the corresponding author on reasonable request.

## Supplementary Materials

The supporting information can be downloaded at: https://www.mdpi.com/article/10.3390/ijms24021236/s1. Reference [47] is cited in the supplementary materials.

## Abbreviations

ABC	ATP-Binding Cassette
ATP	Adenosine Triphosphate
CFTR	Cystic fibrosis transmembrane conductance regulator
DMEM	Dulbecco’s Modified Eagle’s Medium
HEK	Human Embryonic Kidney
ICP	intrahepatic cholestasis of pregnancy
LPAC	low-phospholipid associated cholelithiasis
MSD	Membrane spanning domain
NBD	Nucleotide binding domain
PC	Phosphatidylcholine
PFIC 3	Progressive familial intrahepatic cholestasis type 3
WT	Wild Type
