# Supplementary material for: Ivacaftor-Mediated Potentiation of ABCB4 Missense Mutations Affecting Critical Motifs of the NBDs: Repositioning Perspectives for Hepatobiliary Diseases
Source: Int J Mol Sci. 2023 Jan 8;24(2):1236. doi: 10.3390/ijms24021236 (PMC9867378; doi:10.3390/ijms24021236)
Supplement: Supplementary file 1 [file ijms-24-01236-s001.zip › ijms-2035699-supplementary.pdf]

Ivacaftor-mediated potentiation of ABCB4 missense mutations affecting critical motifs of the NBDs: repositioning perspectives for hepatobiliary diseases

Jean-Louis Delaunay[1], Ahmad Elbahnsi[2], Alix Bruneau[3], Claire Madry[1], Anne-Marie Durand-Schneider[1], Anne Stary[1], Chantal Housset[1,4], Jérémie Gautheron[1], Isabelle Callebaut[2, #], Tounsia Aït-Slimane[1, *, #]

[1]Sorbonne Université, Inserm, Centre de Recherche Saint-Antoine (CRSA), Institute of Cardiometabolism and Nutrition (ICAN), F-75012 Paris, France.

[2]Sorbonne Université, Muséum National d'Histoire Naturelle, UMR CNRS 7590, Institut de Minéralogie, de Physique des Matériaux et de Cosmochimie, IMPMC, 75005, Paris, France

[3]Department of Hepatology & Gastroenterology, Charité Universitätsmedizin Berlin, 13353 Berlin, Germany

[4]Assistance Publique - Hôpitaux de Paris, Hôpital Saint-Antoine, Centre de Référence des Maladies Rares - Maladies Inflammatoires des Voies Biliaires et Hépatites auto-immunes & Service d'Hépatologie, F-75012 Paris, France.

[*] **Corresponding author :** Tounsia Aït-Slimane, PhD—Sorbonne Université, Inserm, Centre de Recherche Saint-Antoine (CRSA) — 27 Rue Chaligny—75012 Paris, France. Phone : +33 (0)1-40-01-13-56. E-mail : tounsia.ait-slimane@inserm.fr

Supplementary Table S1. Human ABCB4 transcript variant A mutagenesis primers

Primer name	*Primer location*	*Primer sequence 5′→ 3′*	*Mutated nucleotide*	*Mutant amino-acid*
Mut 403F	Exon 11	GGAGTTCAATGATGTTCACTTTTCTCACCCTTCTCGAGC	c. 1207 T>C	Y403H
Mut 403R	Exon 11	GCTCGAGAAGGGTGAGAAAAGTGAACATCATTGAACTCC	c. 1207 T>C	Y403H
Mut 435F	Exon 12	TAGTGGCTGTGGGATGAGCACAACGGTCC	c. 1304 A>T	K435M
Mut 435R	Exon 12	GGACCGTTGTGCTCATCCCACAGCCACTA	c. 1304 A>T	K435M
Mut 558F	Exon 14	CCCAAGATCCTTCTGCTGGATAAGGCCACGTCA	c. 1672 G>A	E558K
Mut 558R	Exon 14	TGACGTGGCCTTATCCAGCAGAAGGATCTTGGG	c. 1672 G>A	E558K
Mut 564F	Exon 14	GCCACGTCAGCATTGGGCACAGAAAGTGAAGCT	c. 1691 A>G	D564G
Mut 564R	Exon 14	AGCTTCACTTTCTGTGCCCAATGCTGACGTGGC	c. 1691 A>G	D564G
Mut 589F	Exon 15	CGGACCACCATTGTGATAGCATACCGACTGTCT	c. 1765 C>T	H589Y
Mut 589R	Exon 15	AGACAGTCGGTATGCTATCACAATGGTGGTCCG	c. 1765 C>T	H589Y
Mut 1043F	Exon 25	ATGAAGTCGTGTTCAACCATCCCACCCGAGCAAAC	c. 3127 T>C	Y1043H
Mut 1043R	Exon 25	GTTTGCTCGGGTGGGATGGTTGAACACGACTTCAT	c. 3127 T>C	Y1043H
Mut 1075F	Exon 25	AGTGGCTGTGGGATGAGCACGGTGGTC	c. 3224 A>T	K1075M
Mut 1075R	Exon 25	GACCACCGTGCTCATCCCACAGCCACT	c. 3224 A>T	K1075M
Mut 1200F	Exon 27	AATCCTCCTGTTGGATGCAGCTACATCAGCTCTGG	c. 3599 A>C	E1200A
Mut 1200R	Exon 27	CCAGAGCTGATGTAGCTGCATCCAACAGGAGGATT	c. 3599 A>C	E1200A
Mut 1206F	Exon 27	GAAGCTACATCAGCTCTGGGTACTGAAAGTGAAAAGGTTG	c. 3617 A>G	D1206G
Mut 1206R	Exon 27	CAACCTTTTCACTTTCAGTACCCAGAGCTGATGTAGCTTC	c. 3617 A>G.	D1206G
Mut 1231F	Exon 28	CGCACCTGCATTGTGATTGCTTACCGCCTGTCC	c. 3691 C>T	H1231Y
Mut 1231R	Exon 28	GGACAGGCGGTAAGCAATCACAATGCAGGTGCG	c. 3691 C>T	H1231Y

The underlined nucleotide in the sequence of primers is the mutated base. F represents forward; R, reverse. Mutated nucleotide corresponds to the cDNA of the NM_000443.3 (ABCB4, transcript variant A, mRNA).

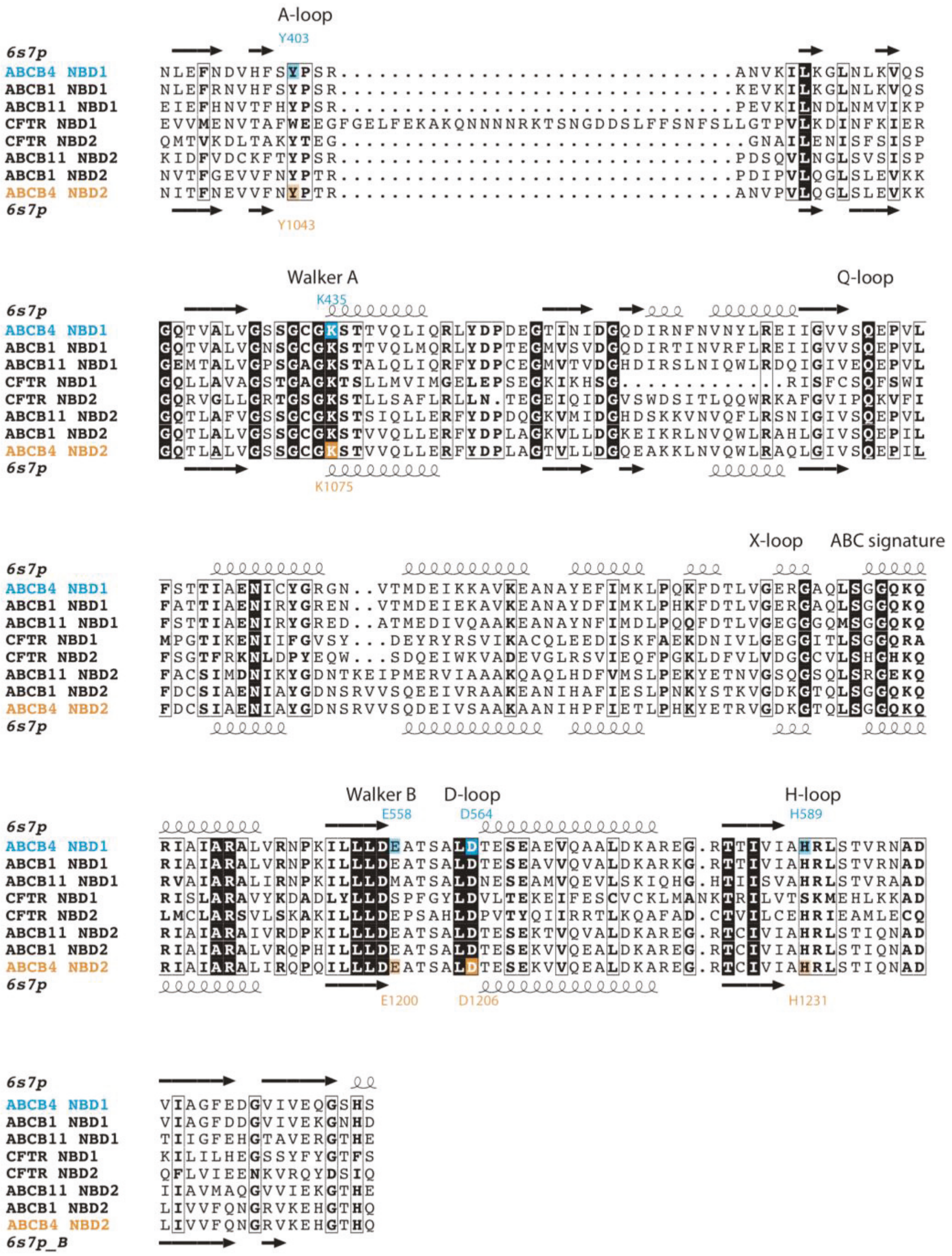

Figure S1. Alignment of the sequences of the nucleotide-binding domains NBD1 and NBD2 of ABCB4 (MDR3 – isoform 2, UniprotP21439-2), ABCB1 (MDR1 – isoform 1, UniprotP08183-1), ABCB11 (BSEP– isoform 1, UniprotPO95342) and CFTR (ABCC7– isoform 1, UniprotP13569). The positions of the ten ABCB4 mutants studied here are shown in blue for NBD1 and orange for NBD2. Note that ABCB4 and ABCB1 have two canonical ATP-binding sites, whereas ABCB11 and CFTR have one degenerated ATP-binding site. The figure was prepared using ESPript (Robert, X. and Gouet, P. 2014)

Reference

Robert, X.; Gouet, P. Deciphering key features in protein structures with the new ENDscript server. *Nucleic Acids Res* **2014**, 42, W320-4, doi: 10.1093/nar/gku316.

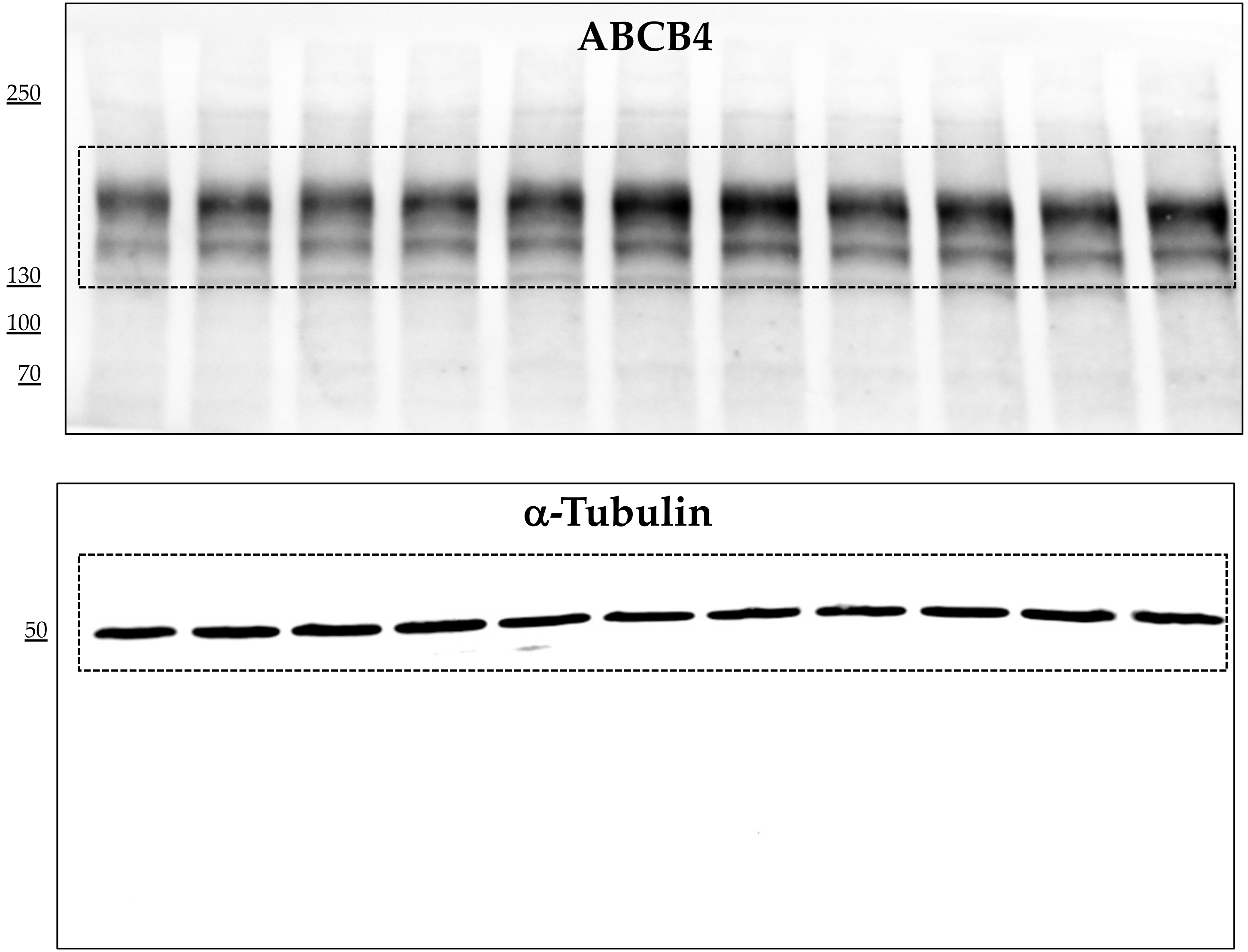

Figure S2. Full immunoblots related to Figure 4A. These immunoblots are representative of three independent experiments. Results shown in Figure 4A are delineated by dotted rectangles. MW (in kDa) are indicated.
